# D’Antona

**Published:** 1883-09

**Authors:** 


### Original Translations.

### ARTICLE XI.

### D'ANTONA.—A CASE OF TOTAL PROLAPSE OF THE RECTUM TREATED BY A NEW OPERATIVE PROCESS.

The prolapse of the rectum was total in a woman seventy years old, was reduceable, but would come down again with an extrusion of 8 centm. The woman was tormented with an intestinal catarrh, and with ulcers here and there upon the mucous membrane, with grave protorrhagias and digestive disorders. Was impossible for her, the erect position; most painful, the walking and sitting.

D'Antona determined on operation, and after anæsthesia, fixed the tumor with four long Billroth's forceps to keep steady both cylinders of the prolapsus, one immovably against the other, and placed a catgut suture, "*a fil passante*," around all the prolapsed tube, 1½ centm. distant from the anal margin. The sewing was double, making a first and a second turn of suture, "*a fil passante*," so that it resulted as if made in a shoemaker sewing manner, otherwise called "in a double row." The aim of this suture was the complete hæmostasia and the preventive reunion of the two ends of the intestine to be recised, in that manner occluding the space of Douglas and of the connective perirectals. Two pins were then fixed ½ centm. anteriorly to the suture to well delimit the circle on which the chassaiqual ecraseur chain had to fall, which, when well tightened, permitted the withdrawal of the pins. When the recision of the prolapsed part was accomplished, the stump retracted powerfully inwards, and, notwithtanding every manœuvre, along with the traction on the four ends of the catgut of the preventive suture, which hanged outside the anus, it became impossible to bring out the recised stump. Nor could it be done, as to dilate the stump with a large tent, which beforehand introduced in the rectum, ligated with a twine, was withdrawn. There was no loss of blood of any account, and another small cotton tent, soaked with a solution of perchloride of iron, was introduced. It clearly results that everything was foreseen in this operation, and every possible inconvenience avoided, for the preventive suture opposed the haemorrhage, the space of Douglas and the connective perirectal was occluded, and therefore the danger of an infection was avoided. The two stumps so remained tightly united and covered at their peritoneal superficies anteriorly, and on the connective behind. Blunted pins with smooth edges were used to prevent the haemorrhage, and were introduced with the guide of the finger as to not involve some intestinal ause, which possibly might have fallen with the prolapsed part. Having made the usual dressing, the patient rested quietly, had no fever, nor suppuration, nor any inconvenience, was perfectly cured at the 15th day.

---

LADY DOCTORS WANTED IN CHINA.—Miss Howard, an American M.D., has for some time practiced her profession in China, where she was fortunate enough to be called to attend the mother of a highly important official, Li Hung Chang, and subsequently the wife also of the same distinguished per-onage. Her fame as a physician has, it is said, spread over all North China, and Miss Howard is now besieged with applications to attend the wives and female relations of wealthy natives, who are entirely averse to consulting a foreign male physician.—*Louisville Med. News.*

THE Cincinnati Board of Health consists of one quack doctor and five saloon-keepers. The health officer is ex-several occupations, so far as we are able to learn, in none successful.—*Lancet and Clinic.*

